# The significance of proline-rich tyrosine kinase2 (Pyk2) on hepatocellular carcinoma progression and recurrence

**DOI:** 10.1038/sj.bjc.6603827

**Published:** 2007-06-05

**Authors:** C K Sun, K T Ng, B S Sun, J W Y Ho, T K Lee, I Ng, R T P Poon, C M Lo, C L Liu, K Man, S T Fan

**Affiliations:** 1 Centre of Cancer Research and Department of Surgery, The University of Hong Kong, Pokfulam, Hong Kong, China; 2 Department of Surgery, Yuquan Hospital, Tsinghua University, Beijing, China; 3 Department of Pathology, The University of Hong Kong, Pokfulam, Hong Kong, China

**Keywords:** hepatocellular carcinoma, recurrence, invasiveness, proline-rich tyrosine kinase2 (Pyk2), disease-free survival

## Abstract

Understanding the precise molecular mechanisms that trigger liver cancer cell migration and invasion could develop novel therapeutic strategies targeting cancer cell invasion to increase the sensitivity to current treatment modalities. In the current study, 49 patients with hepatocellular carcinoma (HCC) were included prospectively. Liver tumour and adjacent non-tumour tissues were detected for the expression of Proline-rich tyrosine kinase 2 (Pyk2), focal adhesion kinase (FAK), ezrin and fibronectin at protein and/or gene levels. Correlation between the expressions of Pyk2/FAK with the clinical pathological data was analysed. Protein expression of Pyk2 was also examined in a nude mice orthotopic liver tumour model with higher metastatic potential. There were 59% (29 out of 49) and 57% (28 out of 49) of HCC patients with higher levels of Pyk2 and FAK protein/gene expression, respectively. We observed a positive correlation between the protein and gene expression levels of Pyk2 and FAK (*P*=0.000, *r*=0.875). Overexpression of Pyk2 and FAK was significantly correlated with shorter disease-free survival. Patients with higher levels of Pyk2/FAK had larger tumour size and advanced Edmonson grading. In the animal studies, Pyk2 overexpression was found in infiltrative tumour cells and lung metastatic nodules. In conclusion, overexpression of Pyk2 and FAK was found in nearly 60% of HCC patients and was significantly correlated with poor prognosis. The significance of Pyk2 in HCC invasiveness was confirmed by animal studies.

Understanding the precise molecular networks that trigger liver cancer cell migration and invasion could develop novel therapeutic strategies targeting cancer cell invasion to increase the sensitivity to current treatment modalities. Focal adhesion kinase (FAK) is an important mediator of cell proliferation, cell survival and migration. Recently, clinical evidences demonstrated that FAK was involved in liver tumour progression and had prognostic significance for hepatocellular carcinoma (HCC) patients (Fujii *et al*, 2004; Itoh *et al*, 2004). In addition to clinical relevance, animal experiments also demonstrated that FAK might play important roles in the regulation of metastatic adhesion of cancer cells with liver sinusoids and formation of organ-specific distant metastases. An *in vitro* study confirmed that FAK integrated growth-factor and integrin signals to promote cell migration (Sieg *et al*, 2000). An *in vivo* animal model also demonstrated that FAK is important for lung metastasis in a breast cancer model (van Nimwegen *et al*, 2005).

Proline-rich tyrosine kinase 2 (Pyk2), also known as cell adhesion kinase*β* (CAK*β*), is a tyrosine kinase that is structurally related to focal adhesion kinase (FAK) (Sasaki *et al*, 1995). Pyk2 has been demonstrated to be able to promote migration and invasion of glioma cells (Lipinski *et al*, 2005) as well as mediate angiogenesis of pulmonary vascular endothelial cells (Tang *et al*, 2002). Moreover, Pyk2 also mediated vascular endothelial cadherin-based cell–cell adhesion and played an important role in the modulation of endothelial integrity (van Buul *et al*, 2005). However, neither expression study nor functional study of Pyk2 in HCC has been reported. It should be worthwhile to explore the potential role of Pyk2 in HCC metastases and recurrence.

Recently, FAK became a potentially important new therapeutic target because of its overexpression in human tumours (McLean *et al*, 2005). To study the expression pattern of Pyk2 and its correlation with clinicopathological data will provide important information for development of novel therapies targeting at focal adhesion kinase family including Pyk2. In the current study, we investigated the correlation between the gene and protein expression levels of Pyk2 and FAK in liver tumour tissues with clinicopathological data, and examined the association of potential metastatic genes (ezrin and fibronectin) with Pyk2. We also explored the significance of Pyk2 in tumour invasiveness and metastases in animal models.

## MATERIALS AND METHODS

### Patients

From December 2000 to October 2001, 49 patients (age over 18 years) diagnosed with HCC that were considered to be resectable on the basis of intraoperative ultrasonography without preoperative adjuvant therapy were included in the current study. The new TNM staging and Edmonson staging of these patients was based on the clinical and pathological diagnosis (Ng *et al*, 2006). The study protocol was approved by the Institutional Review Board of the University of Hong Kong. Informed consent was obtained from each patient before operation.

### Protein expression of Pyk2 and FAK in tumour and adjacent non-tumour liver tissues by immunostaining and Western blot

The paraffin sections of the tissue samples were immunochemically stained for Pyk2 and FAK using Dako EnVision™ system (Dako, Glostrup, Denmark). In brief, after de-paraffinisation, endogenous peroxidase activity was quenched by immersing the sections for 30 min in absolute methanol containing 0.3% H_2_O_2_. The sections were processed to unmask the antigens by conventional microwave oven heating in 10 mM citric acid buffer (pH 6.0) for 12 min. The sections were then treated with 10% normal goat serum for 30 min to reduce the background staining, followed by treatment of appropriate primary antibodies (Pyk2: cytoplasm staining – H-109, Santa Cruz Biotechnology Inc., 2145 Delaware Avenue, Santa Cruz, CA, USA; Nuclear staining – Upstate Biotechnology, Charlottesville, VA, USA; FAK: Upstate Biotechnology) at 4°C overnight. After washing, the sections were incubated with EnVision™ secondary antibody for 30 min at room temperature and then visualised with chromogenic substrate solution for 2 min. The slides were examined under light microscope by two independent investigators with the experience of liver pathology. According to the intensity and area of the staining signalling, the intracellular protein expression of Pyk2 and FAK was classified into higher expression (over 50% of tumour section) and lower expression (less than 50% of tumour section) groups, respectively by an integrated imaging system (MetaMorph Imaging system version 3.0; Universal Imaging Corp, West Chester, PA, USA).

Western blot assay was modified from the method described previously (Liang *et al*, 2003). Proteins from liver tissues were prepared by using urea buffer (8 M urea, pH 8.0). Protein extracts were separated by 12% SDS–PAGE and transferred to PDMF membrane (Millipore, Billerica, MA, USA) according to the standard protocol. After blocking with 5% non-fat milk for 1 h, antibody with proper dilution was hybridised with the membrane at 4°C overnight. The membrane was washed three times with tris buffered saline with tween 20 (TBS/T) each for 10 min and incubated with secondary antibody for 1 h at room temperature. Protein signal was detected by ECL Plus system (Amersham Biosciences, Piscataway, NJ, USA). The signals were quantified by scanning densitometry (Syngene, Cambridge, UK).

### Gene expression of Pyk2, FAK, ezrin and fibronectin in tumour tissues by real-time quantitative RT–PCR

Tissue samples were stored at −80°C until total RNA extraction. The total RNA was extracted using Rneasy Midi Kit (Qiagen, GmbH, Hilden, Germany). About 0.5 *μ*g total RNA from each sample was used to perform reverse transcription (RT) reaction using TaqMan Reverse Transcription Reagents (Man *et al*, 2003) (Applied Biosystems Limited, Foster City, CA, USA). RT product (1 *μ*l) was used to perform real-time quantitative RT–PCR using TaqMan Core Reagent Kit (Applied Biosystems) by the ABI PRISM 7700 Sequence Detection System (Applied Biosystems) (Man *et al*, 2003). The probes and primers of Pyk2, FAK, ezrin and fibronectin were commercially available from Applied Biosystems limited. The TaqMan Ribosomal RNA Control Reagent (18S RNA probe and primer pair; Applied Biosystems) was used for internal control in the same PCR plate well to normalise the target gene amplification copies. All samples were performed in triplicates. The relative gene expression levels were calculated as the ratio of the expression of tumour or non-tumour tissues to normal liver from healthy living donors.

### Functional studies

#### Reagents and plasmids

Plasmids PCDNA3-Pyk2 and PCDNA3-PRNK (C-terminal of Pyk2) were gifts from Dr Joseph Loftus, Mayo Clinic Scottsdale, Scottsdale, USA). PCDNA 3.1 (+) vector was purchased from Invitrogen. Monoclonal antibody against Pyk2 (clone 11) was purchased from BD Transduction Laboratories. Polyclonal antibodies against Phospho-Pyk2 (Tyr402) was purchased from Cell Signalling (Beverly, MA, USA). PLC cells were purchased from ATCC and was grown in Dulbecco's modified Eagle's medium (DMEM) containing 10% FBS, 2 mM L-glutamine, 100 units ml^−1^ streptomycin (Life Technologies, Carlsbad, CA, USA). Metastatic HCC cells MHCC97L was a gift from Dr Tang, Shanghai Fudan University (Lee *et al*, 2005).

#### Cell culture, transfection and stable cell lines

Cells were seeded in six-well plates to 80% confluence. The cells were transfected with 1 *μ*g well^−1^ PCDNA 3.1, PCDNA-Pyk2, PCDNA-PRNK plasmids using Fugene 6 (Roche, Basel, Switzerland). After transfection overnight, cells were changed to normal medium and allowed to recover overnight. The cells were trypsinised and seeded into new 10 cm culture dish and allowed to recover for 2 days. Cells were then grown in DMEM containing G418 at 0.6 mg ml^−1^ until all the non-transfected cells were dead (21 days). Resistant clones were spread in 24-well plates using cloning cylinders and maintained in 0.3 mg ml^−1^ G 418 for further study.

#### Proliferation assay

Cells were trypsinised and counted by using a hemocytometer with 0.2% trypan blue (Life Technologies). Around 5000 cells were seeded onto 96-well plates with DMEM medium supplemented with 10% FBS, 2 mM L-glutamine, 100 units ml^−1^ streptomycin (Life Technologies). 3-(4,5-Dimethylthiazol-2-yl)-2,5-diphenyltetrazolium bromide (MTT) was added at 24 h time interval and signals were measured by a plate reader (BioRad Hercules, CA, USA).

#### Cell invasion assay

Cells were trypsinised and counted in 0.2% trypan blue (Life Technologies). Around 10 000 cells were counted and resuspended in 100 *μ*l of serum-free DMEM medium. Cells were seeded on the upper side of the invasion chamber (BD Biosciences, San Diego, CA, USA). The lower side of the chamber was filled with DMEM supplemented with 10% fetal bovine serum and allowed to incubate at 37°C for 36 h. Cells penetrated through the chamber were stained with 0.1% crystal violet and counted.

#### Extraction of nuclear and cytosolic protein

Nuclear and cytosolic proteins were extracted by ProteoExtract® subcellular proteome extraction kit (Calbiochem, EMD Biosciences Inc., Merck KGaA, Darmstadt, Germany) according to manufacturer's protocol.

#### Immunoblotting

For immunoblotting, fractions were denatured in sample buffer, and resolved in 8–15% SDS-polyacrylamide gel. The proteins were transferred to PVDF membrane (Millipore, Billerica, MA, USA), and immunoblot analysis was performed using antibodies indicated in figure legends. Immune complexes on PVDF were detected by enzyme-linked chemiluminescence (Amersham Biosciences Corp., Piscataway, NJ, USA).

### Animal studies

An orthotopic liver tumour model in nude mice with higher potential of local (intrahepatic) and distant (lung) metastases was established (Lee *et al*, 2005). Briefly, approximately 1 × 10^7^ MHCC97H cells in 0.2 ml culture medium were injected s.c. into the right flank of the mice, which were then observed daily for signs of tumour development. Once the subcutaneous tumour reached 1–1.5 cm in diameter, it was removed and cut into about 1–2 mm cubes which were implanted into the left liver lobe of another group of nude mice. Liver tumour and lung tissues were harvested for immunostaining of Pyk2 at 5 weeks after tumour implantation. The liver tumour and lung metastases were confirmed by H&E staining. The protocol for the *in vivo* animal study has been approved by the Committee on the Use of Live Animals in Teaching and Research (CULATR) of the University of Hong Kong with meeting the standards required by the UKCCCR guidelines. The project number is CULATR-1133-05.

### Statistical analysis

The χ^2^ test was used to compare discrete variables. Mann–Whitney *U*-test was used for statistical comparison for continuous variables. Kaplan–Meier method with log–rank test was used for survival analysis. *P*<0.05 was considered statistically significant. Calculation was made using SPSS computer software version 12 (SPSS, Chicago, IL, USA).

## RESULTS

### Protein and gene expression of Pyk2 and FAK in HCC patients: distinct expression pattern of Pyk2 and FAK

**Figure 1:**
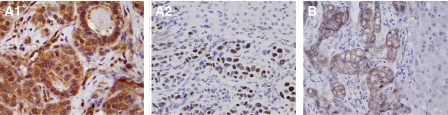
Intracellular protein expression of Pyk2 (**A**) and FAK (**B**). Representative intracellular expression of Pyk2 was found both to be located in the cytoplasm (A1) nuclear of tumour cells (A2). The positive signals of FAK were found in the cytoplasm of tumour cells (**B**).

**Figure 2:**
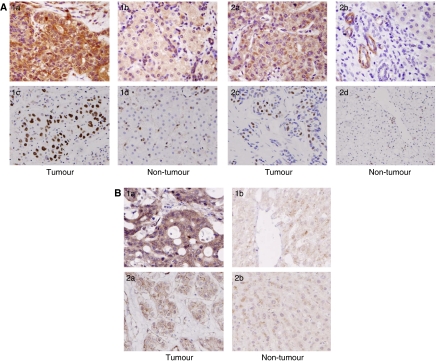
Different expression patterns of Pyk2 (**A**) and FAK (**B**) by immunostaining. There were 59.2% (29 out of 49) of patients with higher intracellular expression levels of Pyk2 in tumour tissues (A1-a/c), whereas 40.8% (20 out of 49) of patients had lower intracellular expression levels of Pyk2 (A2-a/c). There were 57.1% (28 out of 49) of patients with higher intracellular expression levels of FAK in tumour tissues (B1-a), and 42.9% (21 out of 49) of patients had lower intracellular expression levels of FAK in tumour tissues (B2-a). Most of the patients had lower levels of expression of Pyk2 and FAK in non-tumour tissues (B2-b).

**Figure 3:**
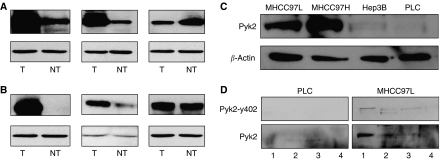
Different protein expression patterns of Pyk2 (**A**) and FAK (**B**) by western blot. PYk2 protein expression in different HCC cell lines (**C**) and subcellular fractionation of Pyk2 in PLC and MHCC97L cell lines (**D**) by Western blot. T: tumour; NT non-tumour. 1: cytoplasmic fraction; Fraction 2: membrane/organelle; Fraction 3: nucleus; Fraction 4: stress fibre.

**Figure 4:**
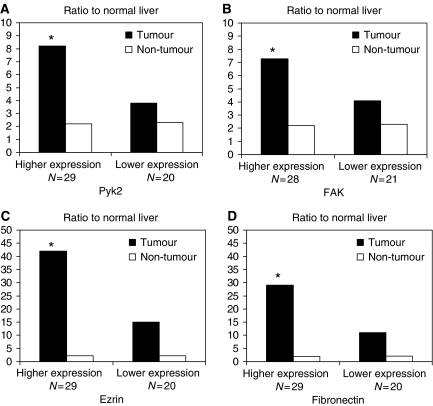
mRNA expression of Pyk2 (**A**), FAK (**B**), Ezrin (**C**) and fibronectin (**D**) in tumour and non-tumour tissues by real-time RT-PCR of patients with higher or lower protein expression of Pyk2 or FAK, respectively. ^*^: Higher expression *vs* lower expression: *P*<0.05.

Different from the previous studies (Stanzione *et al*, 2001; Lipinski *et al*, 2005), positive Pyk2 signals were found both in the cytoplasm and nuclear of tumour cells of HCC patients (Figure 1A). Focal adhesion kinase mainly expressed in the cytoplasm of liver cancer cells (Figure 1B). According to the immunostaining results, there were 29 (59.2%) patients with higher expression levels of Pyk2 and 28 (57%) patients with higher expression of FAK (Figure 2). The protein expression by Western blot was consistent with the immunostaining results (Figure 3). The positive correlation was found between the protein expression levels of Pyk2 and FAK (*r*=0.875, *P*=0.000). The mRNA levels of Pyk2 and FAK detected by quantitative real time RT–PCR were consistent with the intracellular protein expression. Significantly higher mRNA levels of Pyk2 and FAK were found in the tumour tissues of the patients with higher protein expression of Pyk2 and FAK (Figure 4).

### Gene expression of ezrin and fibronectin in HCC patients with higher or lower protein expression of Pyk2

To examine the correlation of Pyk2 with other potential metastatic genes, we detected the mRNA levels of ezrin and fibronectin in the liver tumour and non-tumour tissues. The gene expression levels of ezrin and firbronectin in liver tumour tissues were found consistent with their protein expression level of Pyk2 (Figure 4). Patients with higher protein/gene expression levels of Pyk2 also had higher mRNA levels of ezrin and fibronectin.

### Correlation of the expression of Pyk2 and FAK with clinicopathological parameters: overexpression of Pyk2 was significantly associated with poor survival and tumour invasiveness

Patient survival and pathological data were analysed between the patients with higher or lower protein expression of Pyk2 and FAK in tumour tissues. The higher intracellular protein expression level of Pyk2 was significantly correlated with larger tumour size (*P*=0.005), advanced new Edmonson's staging (*P*=0.033) and venous invasion (*P*=0.02) (Table 1). Similar to the results of Pyk2, overexpression of FAK also contributed to larger tumour size (*P*=0.025) and advanced new Edmonson's staging (*P*=0.017) (Table 2). However, there was no difference in the incidence of venous invasion and advanced tumour-node-metastasis staging between patients with higher and lower expression of FAK.

To further investigate which factors are the potential independent predictors of short survival, univariable and multivariable Cox proportional hazard regression analysis of these factors corresponding to overall and disease-free survival of the HCC patients were also performed. Univariable analysis demonstrated that overexpression of Pyk2/FAK together with other factors were significantly correlated with overall and disease-free survival of the HCC patients after curative resection (Tables 3 and 4). However, multivariable analysis showed that only TNM staging was the independent factor predicting overall and disease-free survival of HCC patients (Tables 3 and 4).

**Figure 5:**
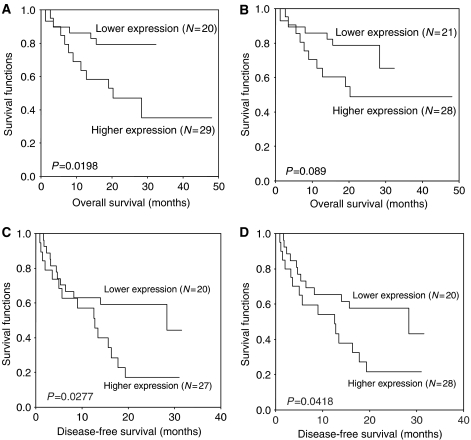
Overall survival comparison between patients with higher and lower expression of Pyk2 (**A**) and FAK (**B**), respectively. Disease-free survival comparison between patients with higher and lower expression of Pyk2 (**C**) and FAK (**D**), respectively.

**Figure 6:**
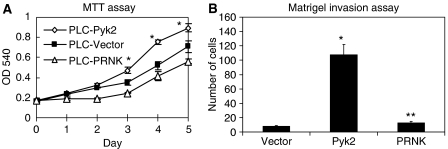
Cell proliferation by MTT assay (**A**) and cell invasion by Matrigel invasion assay (**B**) in PLC cells. (**A**) ^*^P<0.05 – Pyk2 *vs* vector; ^**^P<0.05 – PRNK *vs* Pyk2; (**B**) ^*^P<0.05 Pyk2 *vs* vector and PRNK.

Patients with higher protein expression levels of Pyk2 had significantly lower overall (Figure 5) and disease-free survival (Figure 6) compared to that of patients with lower expression of Pyk2. However, patients with higher protein expression levels of FAK only had lower disease-free survival (Figure 6).

### Expression of Pyk2 in HCC cell lines

To study the expression of Pyk2 in HCC cells, Western blot was performed using monoclonal antibody against Pyk2. Proline-rich tyrosine kinase 2 was expressed in four of the HCC cells (Figure 3C). Metastatic cell lines MHCC97L and MHCC97H have higher expression of Pyk2 as compared to non-metastatic cell lines PLC and Hep3B.

### Distribution of Pyk2 in HCC cells

Previous reports suggest that Pyk2 is localised in the cytoplasm and perinuclear region. To study the distribution of Pyk2 in HCC cells, different protein fractions of the cells were collected and Western blot was performed to study the expression of Pyk2 (Figure 3D). Pyk2 was distributed in cytoplasm and membrane fractions. Nuclear distribution of Pyk2 is also observed in both PLC and MHCC97L cells. The result of this *in vitro* subcellular fractionation was consistent with the *in vivo* intracellular protein expression pattern of Pyk2 (nuclear and cytoplasm staining).

### Pyk2 stimulated proliferation of PLC cells

To characterise further the role of Pyk2 in hepatocellular carcinoma, MTT assay was carried out to study the proliferation of PLC cells tranfected with full-length Pyk2 (Figure 6A). Overexpression of Pyk2 in PLC cells caused reproducible increase in cell growth in presence of 10% serum. As compared to vector control, there was a significant increase in cell proliferation by 25%. Suppression of Pyk2 signalling by PRNK significantly delayed the proliferation of PLC cells in presence of serum. There was a 20% decrease in the rate of proliferation of PLC-PRNK cells as compared to vector control. These results suggested that Pyk2 was involved in the proliferation of PLC cells.

### Pyk2 expression promotes Matrigel invasion

In addition to promotion of cancer cell proliferation, Pyk2 also increased the tumour cell invasiveness detected by the three-dimensional invasion assays. Pyk2 transfectants had higher ability to degrade and move through the matrigel (Figure 6B). Normal PLC cells exhibited the ability to invade the Matrigel. Pyk2 transfectants had 10-fold increased invasion compared to vector control. These results show that Pyk2 promoted an invasive phenotype in PLC cells but not in vector control and PRNK transfectants where the signalling of Pyk2 is being disrupted.

### Protein expression of Pyk2 in liver tumour and lung metastatic nodule in an orthotopic liver tumour model in nude mice: overexpression of Pyk2 correlated with tumour invasiveness and metastasis

**Figure 7:**
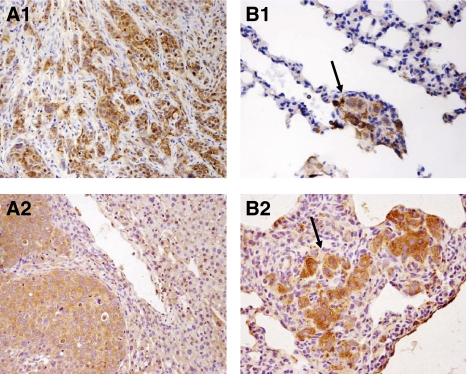
Proline-rich kinase 2 expression of the liver tumour (**A**) and lung metastatic nodule (**B**, arrow) in a nude mice orthotopic liver tumour model with higher potential of local and distant metastases.

To further confirm the significance of Pyk2 in HCC invasiveness, we detected the protein expression of Pyk2 in the liver tumour and metastatic nodules in an orthotopic liver tumour model in nude mice. In the orthotopic liver tumour nude mice model with higher potential of local (intrahepatic) and distant (lung) metastases, strong positive staining of Pyk2 was found in tumour cells with infiltrative growth pattern (Figure 7A). The lung metastatic nodule from the nude mice bearing liver tumour also expressed Pyk2 (Figure 7B). Therefore, the overexpression of Pyk2 was consistent with the aggressive features of the tumour cells.

## DISCUSSION

Development of novel therapies for liver cancer metastases and recurrence is always essential to achieve the long-term survival after curative surgical resection (Tung-Ping *et al*, 2000). Cell migration and invasion are fundamental components of tumour recurrence and metastasis. Understanding the precise molecular mechanism of cancer cell migration and invasion will be important to develop novel therapeutic strategies targeting tumour metastasis and recurrence and to increase the sensitivity of current treatment modalities. Proline-rich tyrosine kinase 2 and FAK play pivotal roles in the regulation of cell motility and invasion (Sasaki *et al*, 1995; Sieg *et al*, 2000; Fujii *et al*, 2004; Itoh *et al*, 2004; van Nimwegen *et al*, 2005). To explore the role of Pyk2 in liver cancer invasiveness, it is necessary to investigate the significance of upregulation of Pyk2 and FAK in tumour recurrence in HCC patients after curative liver resection, and further study the precise function of Pyk2 in tumour metastases.

Significant relevance of overexpression of Pyk2 and disease-free survival was first demonstrated in the current clinical expression study. Consistent with the poor prognosis, patients with higher levels of Pyk2 showed aggressive tumour progression, advanced tumour staging and more invasiveness. Positive correlation between Pyk2 and FAK was also found in our study. In addition to the detection of Pyk2 and FAK in the liver tumour and non-tumour tissues, the gene expression levels of ezrin and fibronectin, two potential metastatic genes, were also investigated according to patients' different expression levels of Pyk2. Ezrin, as a key determinant of tumour metastasis, has been implicated as a conduit for signals between metastasis-associated cell surface molecules and signal transduction components (Hunter, 2004; Khanna *et al*, 2004). Furthermore, recent study demonstrated the co-operative effect of ezrin and c-Src, which also correlated with Pyk2, in cancer cells, in cancer cell metastases (Elliott *et al*, 2004). In this study, higher expression levels of ezrin were found to be consistent with overexpression of Pyk2. Similarly, another gene, fibronectin, which plays important roles in the promotion of cancer cell survival, progression and metastasis, presented higher expression levels in patients with overexpression of Pyk2 (Thant *et al*, 2000; Fornaro *et al*, 2003; Han *et al*, 2006). The association of fibronectin and pyk2 has been reported previously (Loeser *et al*, 2003). The similar expression trend of the genes Pyk2 and FAK with ezrin and fibronectin might also suggest the potential role of Pyk2 in liver cancer metastasis/recurrence.

To confirm our findings in clinical samples, we further conducted *in vitro* functional studies and an *in vivo* animal study in an orthotopic liver tumour model with highly local (intrahepatic) and distant (lung) metastatic potential. Our animal study also first demonstrated that overexpression of Pyk2 was found in infiltrative liver tumour cells (local metastasis) and lung metastatic nodules. This *in vivo* animal study further confirmed the significant correlation of Pyk2 with HCC invasiveness. In addition to animal study, our primary function study also indicated that Pyk2 played important role in HCC cell proliferation and invasion. However, the distinct cell signalling pathways under the regulation of Pyk2 related to cell invasion and migration should be investigated to elucidate the exact molecular mechanism in addition to the phenomenon demonstrated in our clinical and animal studies.

In summary, overexpression of Pyk2 and FAK was found in nearly 60% of HCC patients and was significantly correlated with poor prognosis. The significance of Pyk2 in HCC invasiveness was also demonstrated by our orthotopic liver cancer animal model with higher metastatic potential. Therefore, Pyk2 would be a novel therapeutic target as well as a prognostic marker in addition to FAK. Functional studies will be worthwhile for investigation of its role in liver cancer recurrence and metastases. To examine the precise molecular mechanism of Pyk2 in liver cancer metastasis and the possible pathways involved, we are currently conducting a series of *in vitro* and *in vivo* studies. Further studies will be needed to explore the potential therapies targeting at Pyk2 in liver cancer recurrence and metastases.

## Figures and Tables

**Table 1:** Correlation with clinicopathological parameters–Pyk2

	**Higher expression (*n*=29)**	**Lower expression (*n*=20)**	***P*-value**
Tumour size (cm)	9 (2.5–16)	3.5 (2.3–12)	*0.005*
New Edmonson grading (III, IV)	26/29 (89.7%)	12/20 (60%)	*0.033*
Venous invasion	17/29 (58.6%)	5/20 (25%)	*0.02*
New TNM staging (III, IV)	10/29 (34.5%)	3/20 (15%)	0.191

Abbreviations: Pyk2=proline-rich tyrosine kinase 2; TNM=tumour-node-metastasis.

**Table 2:** Correlation with clinicopathological parameters–FAK

	**Higher expression (*n*=28)**	**Lower expression (*n*=21)**	***P*-value**
Tumour size (cm)	8.2 (2.5–16)	3.5 (2.3–15)	*0.025*
New Edmonson grading (III, IV)	25/28 (89.2%)	12/21 (57.1%)	*0.017*
Venous invasion	15/28 (53.6%)	7/21 (33.3%)	0.159
New TNM staging (III, IV)	9/28 (32.1%)	4/21 (19%)	0.348

Abbreviations: FAK=focal adhesion kinase; TNM=tumour-node-metastasis.

**Table 3:** Cox proportional hazard regression analysis of Pyk2 protein expression and clinicopathological parameters in relation to the overall survival of HCC patients

	**Univariable analysis**		**Multivariable analysis**	
	**HR (95% CI)**	***P***	**HR (95% CI)**	***P***
Pyk2 protein	4.63 (1.5–14.31)	0.008	3.19 (0.48–21.22)	NS
Higher *vs* lower expression				
FAK protein	3.18 (1.17–8.63)	0.023	2.16 (0.44–10.57)	NS
Higher *vs* lower expression				
New TNM staging	2.09 (1.49–2.93)	0.000	2.42 (1.37–4.23)	0.002
Advanced *vs* early staging				
Venous invasion	2.97 (1.38–6.41)	0.006	0.4 (0.1–1.64)	NS
Presence *vs* absence				
New Edmonson staging	1.08 (0.95–1.23)	0.226	1.23 (0.94–1.63)	NS
Advanced *vs* early staging				

Abbreviations: CI=confidence interval; FAK=focal adhesion kinase; HCC=hepatocellular carcinoma; HR=hazard ratio; NS=not significant; Pyk2=proline-rich tyrosine kinase 2; TNM=tumour-node-metastasis.

**Table 4:** Cox proportional hazard regression analysis of Pyk2 protein expression and clinicopathological parameters in relation to the disease free survival of HCC patients

	**Univariable analysis**		**Multivariable analysis**	
	**HR (95% CI)**	***P***	**HR (95% CI)**	***P***
Pyk2 protein	5.33 (2.27–14.48)	0.000	2.57 (0.7–9.54)	NS
Higher *vs* lower expression				
FAK protein	3.29 (1.51–7.17)	0.003	2.42 (0.59–9.87)	NS
Higher *vs* lower expression				
New TNM staging	1.61 (1.24–2.93)	0.000	1.87 (1.03–3.36)	0.039
Advanced *vs* early staging				
Venous invasion	2.68 (1.49–4.82)	0.001	1.35 (0.46–4.02)	NS
Presence *vs* absence				
New Edmonson staging	1 (0.89–1.12)	0.975	1.12 (0.77–1.35)	NS
Advanced *vs* early staging				

CI=confidence interval; HR=hazard ratio; NS=not significant.
